# Toll-Like Receptor Polymorphisms and Susceptibility to Urinary Tract Infections in Adult Women

**DOI:** 10.1371/journal.pone.0005990

**Published:** 2009-06-22

**Authors:** Thomas R. Hawn, Delia Scholes, Shuying S. Li, Hongwei Wang, Yin Yang, Pacita L. Roberts, Ann E. Stapleton, Marta Janer, Alan Aderem, Walter E. Stamm, Lue Ping Zhao, Thomas M. Hooton

**Affiliations:** 1 Department of Medicine, University of Washington, Seattle, Washington, United States of America; 2 Department of Epidemiology, University of Washington, and Center for Health Studies, Group Health Cooperative, Seattle, Washington, United States of America; 3 Fred Hutchinson Cancer Research Center, Seattle, Washington, United States of America; 4 Institute for Systems Biology, Seattle, Washington, United States of America; 5 Department of Medicine, University of Miami, Miami, Florida, United States of America; Institut Pasteur, France

## Abstract

**Background:**

Although behavioral risk factors are strongly associated with urinary tract infection (UTI) risk, the role of genetics in acquiring this disease is poorly understood.

**Methodology/Principal Findings:**

To test the hypothesis that polymorphisms in Toll-like receptor (TLR) pathway genes are associated with susceptibility to UTIs, we conducted a population-based case-control study of women ages 18–49 years. We examined DNA variants in 9 TLR pathway genes in 431 recurrent cystitis (rUTI) cases, 400 pyelonephritis cases, and 430 controls with no history of UTIs. In the Caucasian subgroup of 987 women, polymorphism TLR4_A896G was associated with protection from rUTI, but not pyelonephritis, with an odds ratio (OR) of 0.54 and a 95% confidence interval (CI) of 0.31 to 0.96. Polymorphism TLR5_C1174T, which encodes a variant that abrogates flagellin-induced signaling, was associated with an increased risk of rUTI (OR(95%CI): 1.81 (1.00–3.08)), but not pyelonephritis. Polymorphism TLR1_G1805T was associated with protection from pyelonephritis (OR(95%CI): 0.53 (0.29–0.96)).

**Conclusions:**

These results provide the first evidence of associations of TLR5 and TLR1 variants with altered risks of acquiring rUTI and pyelonephritis, respectively. Although these data suggest that TLR polymorphisms are associated with adult susceptibility to UTIs, the statistical significance was modest and will require further study including validation with independent cohorts.

## Introduction

Acute uncomplicated urinary tract infections (UTIs) in young women are exceedingly common and result in substantial morbidity, time lost from work, and medical costs. Treatment requires the frequent use of antibiotics which contributes to drug resistance. Recurrent UTI (rUTI) is a common syndrome in otherwise young healthy women. Previous studies suggest that 27% to 44% of women who experience an initial UTI develop rUTI [1], [2]. The vast majority of these women do not have underlying functional or anatomic abnormalities of the urinary tract. Although pyelonephritis is less common than cystitis, it is a serious illness that can result in expensive hospitalization. Behavioral factors, such as sexual intercourse and spermicide use, are strongly associated with an increased risk of rUTI and pyelonephritis [3], [4], [5]. However, many women with uncomplicated UTI do not have obvious behavioral, functional or anatomic risk factors, suggesting that genetic risk factors may be present.

A series of studies over several decades indicates that host genetic factors influence susceptibility to human infections [6], [7], [8]. More recent studies suggest an influence of genetics on susceptibility to UTIs. In one family study, 15% of relatives of pyelonephritis-prone children had a UTI history compared to 3% of relatives of controls [9]. In adults, 65.5% of mothers, 60.7% of daughters, and 48.6% of sisters of women with rUTI had a similar history [10]. We previously found that adult women with rUTI or pyelonephritis were more likely to have a mother with a UTI history in comparison to controls [4], [5]. Aside from associations of non-secretor blood group antigens and P1 phenotype with RUTI and/or pyelonephritis, we are not aware of any associations of polymorphisms with UTIs in adults [11], [12], [13], [14], [15], [16]. Genetic studies in children have reported associations of polymorphisms in CXCR1, TLR2, and TLR4 with UTI susceptibility [17], [18], [19], [20]. In addition, reduced expression levels of CXCR1, CXCR2 and TLR4 on neutrophils was associated with pyelonephritis, recurrent cystitis and asymptomatic bacteriuria, respectively [18], [21], [22], [23]. Mouse studies have also suggested a role for CXCR1 in UTI susceptibility [21]. Although these studies suggest a possible role for genetics in human UTI susceptibility, the genes involved remain largely unknown.

Toll-like receptors (TLRs) are a family of germline-encoded receptors that orchestrate the innate immune response and recognize Pathogen-Associated Molecular Patterns (PAMPs) such as bacterial flagellin (TLR5), lipopolysaccharide (LPS) (TLR4), and bacterial lipopeptides (TLR1/2/6) [24], [25], [26]. During a UTI, bacteria colonize the urethra and ascend to the bladder, where they can persist at high levels and cause cystitis [27]. In addition, pathogens may ascend to the kidney and cause serious complications, including pyelonephritis and bacteremia [28]. The initial recognition of bacteria occurs at the epithelial cell surface of the urogenital tract, a site of TLR expression in humans [29]. *E. coli*, which causes 70–90% of all uncomplicated UTIs, is recognized by several TLRs, including TLR1,2,4,5,6 and 11 [24], [25], [26]. Although previous studies in mice indicate that TLR4, TLR5, and TLR11 regulate susceptibility to cystitis and pyelonephritis, the role of TLRs in human UTI pathogenesis is poorly understood [30], [31], [32], [33].

We and others have characterized human TLR pathway polymorphisms that are associated with altered gene function and susceptibility to different infections [34], [35], [36], [37], [38], [39], [40], [41], [42], [43]. Although two previous studies suggest that polymorphisms TLR2_G2258A and TLR4_A896G are associated with susceptibility to UTIs in children, the role of the other functionally significant TLR polymorphisms in UTI pathogenesis is not currently known [19], [20]. In addition, it is also not known whether any TLR variants are associated with cystitis or pyelonephritis in adults. In this manuscript, we summarize the results of a population-based case-control study examining whether polymorphisms in TLR genes are associated with susceptibility to cystitis and pyelonephritis in adult women ages 18–49 years.

## Materials and Methods

### Study Setting and Participants

The study protocols were approved by the Human Subjects committees at Group Health Cooperative, the University of Washington, and Western Institutional Review Board. The study was conducted at Group Health Cooperative in Seattle, Washington. We selected potential RUTI and pyelonephritis cases from the health plan's automated databases. Potential cystitis subjects were identified through having received an International Classification of Diseases (ICD-9) diagnosis code. Recurrent cystitis (rUTI) case subjects were identified based on 3 diagnosed UTI episodes within a 12-month time frame or 2 UTIs within 6 months (episodes were separated by at least 30 days). Culture confirmation (≥10^3^ cfu/mL of a urinary pathogen) or UTI guideline-related treatment was required for all UTI episodes in the cluster. Potential pyelonephritis subjects were identified through having received a pyelonephritis ICD-9 diagnosis code and, if they received only outpatient treatment, a primary diagnosis of pyelonephritis and an accompanying culture result of ≥10^3^ cfu/mL of a urinary pathogen or accompanying antibiotic therapy appropriate for pyelonephritis. The remainder of the women in the registries constituted the potential control subjects who were randomly selected and frequency-matched by case age group (age categories were 18–29, 30–39, 40–49 years).

Potential participants received a letter of invitation describing the study and inviting their participation. In screening for eligibility, exclusion criteria were kept to a minimum. Potential participants were queried as to whether they could urinate on their own and were ambulatory. In addition, potential control subjects were asked about previous UTIs and were excluded if they reported previous healthcare provider-diagnosed cystitis episodes. If they reported a history of pyelonephritis or kidney infections, they were enrolled in the pyelonephritis case group. Women who were eligible and willing to take part in the study were scheduled for a clinic visit where case histories of recurrent cystitis and/or pyelonephritis were confirmed. The number of lifetime UTIs was determined by self-reporting. For controls, we verified that they had no history of UTIs. Ethnicity was determined by self-identification. We identified 877 women as potential rUTI subjects, 673 as potential pyelonephritis subjects, and 1,923 as potential control women during recruitment. Of the potential rUTI case participants, 431 (59%) of women identified as eligible agreed to participate and completed their clinic appointments; 144 were identified as ineligible; 256 refused; and 46 could not be reached. Among potential pyelonephritis case women, 400 (69%) of women identified as eligible agreed and completed clinic appointments; 89 were ineligible; 155 refused; and 29 could not be reached. Of the identified potential control women, 430 (47%) of identified eligible women agreed and made clinic visits; 999 were ineligible, due in large part to having a history of cystitis; 440 refused; and 54 could not be reached.

#### Genomic techniques

Genomic DNA was purified from peripheral blood by QIAamp DNA blood kit (Qiagen). For TLR2, 4, 5, MYD88 (Myeloid differentiation primary response gene 88), TIRAP/MAL (TIR domain containing adapter/MYD88 adaptor-like), TICAM1/TRIF (TIR-Domain-containing adaptor molecule 1/TIR domain-containing adaptor inducing interferon beta), and TRAM (TRIF-related adaptor molecule), we sequenced the coding region to look for polymorphisms. We attempted to sequence 48 or 96 samples per gene depending on levels of previous investigations. We obtained high quality sequence of the entire coding region in 45 subjects for TLR2, 43 for TLR4, 46 for TLR5, 86 for TIRAP, and 87 for TRIF. We amplified the coding region by PCR, sequenced it with Big Dye Terminator v3.0 and then analyzed it on an ABI PRISM 3730 capillary sequencer (Applied Biosystems). Sequence was aligned and analyzed with the programs PHRED/PHRAP and CONSED [44]. For genotyping in the full cohort, we generated haplotype tagging SNPs from our sequencing data as well as publicly available data from the Innate Immunity Program in Genomic Applications (IIPGA, http://innateimmunity.net/). For determining haplotype tagging SNPs, we used a multilocus linkage disequilibrium measure based on generalized mutual information,which is also known as relative entropy or Kullback-Leibler distance [45]. Genotyping was carried out with a MassARRAY™ technique (Sequenom) as previously described [46], [47].

#### Statistical Analyses

We evaluated the associations of case-control status (the outcome) and SNP genotypes under log-additive, recessive, and dominant models. In the log-additive model (also called an allelic trend test), common homozygous genotypes (00) were assigned a value of 0, heterozygotes (01) a value of 1, and minor homozygous genotypes (11) a value of 2. Odds ratios and significance levels were then assessed using a logistic regression model. For the dominant model analysis, we combined genotypes 01 and 11 and compared to genotype 00. For the recessive model, we compared genotypes 00 and 01 versus genotype 11. We also evaluated the association of case-control status with haplotypes of SNPs in constructed with an Expectation/Maximization (EM) algorithm with the program HPlus as previously described [48]. We analyzed the rUTI and pyelonephritis cases separately and in combination. Two-sided testing was used for all comparisons to evaluate statistical significance, considering a P-value of ≤0.05 as significant. We evaluated the associations of SNPs with UTI intensity using a linear regression model for each case group and combined case groups. The coefficient with respect to SNP represents increased/decreased UTI intensity. The significance of coefficient measures the strength of the association of SNP genotypes with UTI intensity. To verify that our significant findings were not due to population admixture, we also performed Caucasian subgroup analyses. All analyses were performed using the software Hplus or SAS [48]. Hardy-Weinberg equilibrium (HWE) testing was performed for all SNP genotypes using Haploview. Except for minor deviations, all SNP genotypes satisfied Hardy-Weinberg in the Caucasian control group.

## Results

### TLR polymorphism discovery

We used a case-control study design to examine whether TLR pathway gene polymorphisms were associated with susceptibility to recurrent cystitis and/or pyelonephritis in adult women. Cases and controls had a similar mean age and ethnic composition and were generally healthy with minimal co-morbid conditions (Table 1). We selected 9 genes for analysis, including TLRs 1, 2, 4, 5, 6 due to their central role in recognizing *E. coli* and other UTI-associated pathogens. We also chose to examine the adaptor molecules associated with these TLRs, including MYD88, TIRAP, TRIF, and TRAM. In order to discover novel polymorphisms associated with UTIs, we PCR-amplified and sequenced the coding regions of 7 TLR pathway genes (TLR2, TLR4, TLR5, MYD88, TIRAP, TRIF, and TRAM) in subjects with a high frequency of cystitis or pyelonephritis episodes. We did not discover any polymorphisms at >2% frequency in MYD88 or TRAM, so these genes were not studied further. We discovered previously reported polymorphisms in TLR2 (n = 45 subjects), TLR4 (n = 43), TIRAP (n = 86), and TRIF (n = 87) in this study population. In TLR5, among 46 case women, we found several previously reported polymorphisms as well as two novel SNPs (C541A (Q181K), ss136261639 in the NCBI dbSNP database (http://www.ncbi.nlm.nih.gov/SNP/) in 4/46 and A2254G (R752G), ss136261646 in 1/40). Together, the results for these 5 genes suggest that nearly all polymorphisms were previously available in public databases for genotyping strategies.

**Table 1:** Characteristics of Cases and Controls.

Variable	rUTI cases (N = 431)	Pyelo cases (N = 400)	Controls (N = 430)
	N (%)*	N (%)*	N (%)*
Age at study enrollment, years (mean)	37.6	36.7	37.5
Ethnicity			
American Indian/Alaska native	16 (3.7)	22 (5.5)	13 (3.0)
Asian	56 (13.0)	25 (6.3)	60 (14.0)
Black or African American	24 (5.6)	30 (7.5)	32 (7.4)
Native Hawaiian or Pacific Is.	7 (1.6)	9 (2.3)	10 (2.3)
Caucasian	339 (78.7)	321 (80.3)	317 (73.7)
Hispanic/Latino	29 (6.7)	40 (10.0)	26 (6.1)
Other	20 (4.6)	26 (6.5)	20 (4.7)
Health conditions (history of)			
Kidney stones	13 (3.0)	46 (11.5)	5 (1.2)
Kidney failure/insufficiency	1 (0.2)	5 (1.3)	2 (0.5)
Diabetes (not during pregnancy)	8 (1.9)	23 (5.8)	10 (2.3)
Urinary Tract Procedure History			
Bladder/kidney surgery	17 (3.9)	40 (10.0)	5 (1.2)
Cystoscopy	33 (7.8)	48 (12.1)	0

* Numbers and percentages in ethnicity subcategories can be greater than total number due to selection of more than one category for an individual.

For our primary analysis, we examined whether 7 well-characterized TLR-pathway SNPs were associated with UTIs. Previous studies suggest that these SNPs are associated with altered TLR gene function and susceptibility to different infections. The polymorphisms include TLR1_G1805T (amino acid (AA) change S602I), TLR2_G2258A (AA R753Q), TLR4_A896G (AA D299G), TLR4_C1196T (AA T399I), TLR5_C1174T (AA R392STOP), TIRAP_C539T (AAS180L), and TIRAP_C558T (AA A186A) [34], [35], [36], [37], [38], [39], [40], [41], [42], [43]. Although TIRAP_C558T is a synonymous SNP, we have previously shown that it is associated with altered cytokine production in response to PAM2 stimulation [36]. We compared allele and genotype frequencies between cases and controls and found associations between SNPs TLR5_C1174T, TLR4_A896G, and TLR1_G1805T with UTI outcomes. Due to the presence of population heterogeneity in the entire cohort, we analyzed data in both the Caucasian subgroup as well as the entire cohort (Tables 2–3 for Caucasian subgroup and Supplemental Tables 1 and 2 for the entire cohort). The ethnic composition of the case and control populations was similar with a predominance of a Caucasian background in all groups (Caucasian frequency of 78.7% in RUTI cases, 80.3% in pyelonephritis cases, and 73.7% in controls, Table 1).

**Table 2:** Genotypic Analysis of Functional TLR SNPs in Caucasian Subgroup.

SNP	group	genotype			Log-additive Model		Recessive Model 11 vs 00, 01		Dominant Model 01, 11 vs 00	
		00	01	11	OR (95% CI)	*P* a	OR (95%CI)	*P*	OR (95%CI)	*P*
TLR1	Control	139 (48.6)	114 (39.9)	33 (11.5)						
G1805T	rUTI	150 (49.5)	120 (39.6)	33 (10.9)	0.97 (0.76, 1.23)	0.781	0.94 (0.56, 1.56)	0.803	0.96 (0.70, 1.33)	0.827
	Pyelo	134 (47.7)	129(45.9)	18 (6.4)	0.78 (0.55, 1.10)	0.158	0.53 (0.29, 0.96)	**0.035**	1.04 (0.75, 1.44)	0.828
	Combined	284(48.63)	249 (42.6)	51 (8.7)	0.72 (0.53, 0.97)	**0.033**	0.73 (0.46, 1.17)	0.190	1.00 (0.75, 1.33)	0.994
TLR2	Control	297 (93.99)	19 (6.01)	0						
G2258A	rUTI	320 (94.96)	17 (5.04)	0	0.83 (0.42, 1.63)	0.588			0.83 (0.42, 1.63)	0.588
	Pyelo	302 (94.38)	18 (5.63)	0	0.93 (0.48, 1.81)	0.835			0.93 (0.48, 1.81)	0.835
	Combined	622 (94.67)	35 (5.33)	0	0.88 (0.50, 1.56)	0.662			0.88 (0.50, 1.56)	0.662
TLR4	Control	274 (87.5)	33 (10.5)	6(1.9)						
A896G	rUTI	310 (92.8)	23 (6.9)	1 (0.3)	0.54 (0.31, 0.96)	**0.035**	0.15 (0.02, 1.28)	0.084	0.54 (0.32, 0.93)	**0.025**
	Pyelo	275 (47.7)	42(45.9)	1 (6.4)	0.94 (0.57, 1.57)	0.816	0.16 (0.02, 1.35)	0.092	1.10 (0.69, 1.75)	0.692
	Combined	585 (89.7)	65 (10.0)	2 (0.3)	0.72 (0.46, 1.15)	0.169	0.16 (0.03, 0.79)	0.024	0.81 (0.53, 1.23)	0.311
TLR4	Control	277 (87.66)	35 (11.08)	4 (1.27)						
C1196T	rUTI	312 (92.31)	26 (7.69)	0	0.66 (0.39, 1.12)	0.126			0.59 (0.35, 0.998)	0.049
	Pyelo	277 (86.56)	43 (13.44)	0	1.23 (0.76, 1.98)	0.397			1.10 (0.69, 1.75)	0.680
	Combined	589 (89.51)	69 (10.49)	0	0.93 (0.60, 1.43)	0.731			0.83 (0.55, 1.26)	0.388
TLR5	Control	292 (92.7)	21 (6.8)	2(0.6)						
C1174T	rUTI	295 (87.5)	41 (12.2)	1 (0.3)	1.65 (0.89, 3.05)	0.110	0.47 (0.04, 5.16)	0.534	1.81 (1.00, 3.08)	**0.030**
	Pyelo	293(91.6)	26 (8.1)	1 (0.3)	1.06 (0.57, 1.97)	0.848	0.49 (0.04, 5.44)	0.562	1.17 (0.17, 2.09)	0.596
	Combined	588 (89.5)	67 (10.2)	2 (0.3)	1.37 (0.78, 2.41)	0.275	0.48 (0.07, 3.41)	0.461	1.49 (0.91, 2.44)	0.113
TIRAP	Control	225 (71.66)	79 (25.16)	10 (3.18)						
C539T	rUTI	251 (74.26)	77 (22.78)	10 (2.96)	0.87 (0.61, 1.25)	0.464	0.93 (0.38, 2.26)	0.867	0.88 (0.62, 1.24)	0.454
	Pyelo	237 (74.53)	75 (23.58)	6 (1.89)	0.57 (0.20, 1.59)	0.284	0.59 (0.21, 1.63)	0.304	0.86 (0.61, 1.23)	0.416
	Combined	488 (74.39)	152 (23.17)	16 (2.44)	0.89 (0.65, 1.22)	0.455	0.76 (0.34, 1.69)	0.502	0.87 (0.64, 1.18)	0.367
TIRAP	Control	192 (61.34)	107 (34.19)	14 (4.47)						
C558T	rUTI	181 (55.02)	131 (39.82)	17 (5.17)	1.30 (0.94, 1.80)	0.116	1.16 (0.56, 2.40)	0.683	1.30 (0.95, 1.78)	0.105
	Pyelo	190 (59.94)	110 (34.70)	17 (5.36)	1.23 (0.59, 2.56)	0.587	1.21 (0.59, 2.50)	0.607	1.06 (0.77, 1.46)	0.718
	Combined	371 (57.43)	241 (37.31)	34 (5.26)	1.17 (0.88, 1.55)	0.295	1.19 (0.63, 2.25)	0.599	1.18 (0.89, 1.55)	0.249

a P-value<0.05 is in bold.

**Table 3:** TLR Polymorphisms & Association with UTI Disease Status in Caucasian Subgroup.

Gene	SNP a	Allele	HWE b	Minor Allele Frequency				rUTI vs Control		Pyelo vs Control		Combined vs Control	
				Control (317)	RUTI (339)	Pyelo (321)	Combined (660)	OR, 95% CI	P	OR, 95% CI	P	OR, 95% CI	P
TLR1	zC6003T	T/C	0.528	0.19	0.18	0.18	0.18	0.98 (0.74, 1.30)	0.876	0.95 (0.71, 1.26)	0.715	0.96 (0.75, 1.23)	0.764
	zC6165T(rs5743594)	C/T	0.622	0.19	0.21	0.20	0.20	1.13 (0.86, 1.48)	0.391	1.07 (0.81, 1.41)	0.657	1.10 (0.86, 1.40)	0.451
	T130C	T/C	1.000	0.00	0.03	0.00							
	G239C(rs5743611)	G/C	1.000	0.10	0.08	0.09	0.09	0.81 (0.55, 1.20)	0.286	0.94 (0.64, 1.38)	0.741	0.87 (0.62, 1.22)	0.415
	A743G (rs4833095)	A/G	1.000	0.27	0.27	0.26	0.27	1.00 (0.78, 1.28)	0.985	0.95 (0.74, 1.22)	0.676	0.98 (0.79, 1.21)	0.825
	G1805T (rs5743618)	G/T	1.000	0.31	0.31	0.29	0.30	0.96 (0.75, 1.23)	0.773	0.91 (0.70, 1.17)	0.441	0.94 (0.75, 1.16)	0.547
TLR2	zT540A (rs4696480)	T/A	7.00E-04	0.45	0.49	0.45	0.47	1.10 (0.90, 1.39)	0.191	0.99 (0.80, 1.05)	0.925	1.03 (0.91, 1.22)	0.303
	C597T (rs3804099)	T/C	0.985	0.43	0.46	0.44	0.45	1.13 (0.91, 1.41)	0.276	1.06 90.85, 1.33)	0.588	1.10 (0.91, 1.33)	0.345
	T1350C (rs3804100)	T/C	1.000	0.07	0.09	0.08	0.09	1.35 90.91, 2.01)	0.141	1.16 (0.77, 1.76)	0.480	1.26 (0.88, 1.80)	0.210
	C1892A (rs5743704)	C/A	7.19E-13	0.04	0.04	0.03	0.04	1.11 (0.62, 1.98)	0.729	0.98 (0.54, 1.80)	0.957	1.05 (0.63, 1.75)	0.861
	G2258A (rs5743708)	G/A	2.17E-09	0.03	0.03	0.03	0.03	0.84 (0.43, 1.62)	0.594	0.93 (0.49, 1.80)	0.837	0.88 (0.50, 1.56)	0.667
	zG1868A (rs1898830)	A/G	0.788	0.33	0.33	0.34	0.33	0.92 90.50, 1.39)	0.883	0.95 (0.66, 1.38)	0.982	0.96 (0.68, 1.32)	0.895
TLR4	zA11547G	A/G	0.154	0.08	0.11	0.13	0.12	1.31 (0.90, 1.90)	0.155	1.58 (1.10, 2.28)	0.014	1.44 (1.04, 2.00)	0.029
	zG11912T (rs2149356)	G/T	0.249	0.25	0.28	0.30	0.29	1.16 (0.89, 1.53)	0.272	1.25 (0.95, 1.63)	0.108	1.21 (0.95, 1.53)	0.122
	zG11995A	G/A	0.013	0.33	0.31	0.32	0.32	0.91 (0.72, 1.15)	0.412	0.97 (0.76, 1.22)	0.780	0.94 (0.76, 1.15)	0.522
	T315C (rs5030710)	T/C	1.000	0.00	0.05	0.02	0.03						
	A896G (rs4986790)	A/G	0.931	0.07	0.04	0.07	0.05	0.50 (0.30, 0.83)	0.007	0.96 (0.62, 1.48)	0.851	0.72 (0.49, 1.06)	0.099
	C1196T (rs4986791)	C/T	0.003	0.07	0.04	0.07	0.05	0.55 (0.33, 0.90)	0.018	0.99 (0.64, 1.53)	0.952	0.76 (0.51, 1.12)	0.167
	GP3124C	G/C	0.705	0.15	0.15	0.14	0.14	0.96 (0.71, 1.31)	0.803	0.90 (0.66, 1.23)	0.499	0.93 (0.71, 1.22)	0.597
	zA17923C	C/A	0.041	0.09	0.11	0.13	0.12	1.28 (0.78, 2.09)	0.326	1.55 (0.94, 2.54)	0.085	1.40 (0.90, 2.18)	0.138
TLR5	C541A	C/A	1.000	0.03	0.05	0.03	0.04	1.61 (0.87, 2.97)	0.129	1.05 (0.54, 2.07)	0.880	1.33 (0.76, 2.35)	0.319
	C1174T (rs5744168)	C/T	1.000	0.04	0.06	0.04	0.05	1.65 (1.00, 2.73)	0.052	1.11 (0.64, 1.92)	0.718	1.38 (0.87, 2.20)	0.173
	A1775G (rs2072493)	A/G	0.391	0.16	0.18	0.15	0.16	1.09 (0.82, 1.46)	0.559	0.89 (0.66, 1.21)	0.454	0.99 (0.77, 1.28)	0.949
	T1846C (rs5744174)	T/C	0.443	0.37	0.38	0.42	0.40	1.05 (0.84, 1.32)	0.660	1.20 (0.96, 1.51)	0.116	1.12 (0.92, 1.37)	0.251
	A2254G	A/G	1.000	0.02	0.02	0.00	0.00	0.94 (0.06, 14.9)	0.962			0.48 (0.03, 7.68)	0.604
	zA35403G (rs1053954)	A/G	1.000	0.11	0.12	0.14	0.13	1.07 (0.76, 1.51)	0.711	1.27 (0.90, 1.78)	0.175	1.16 (0.86, 1.57)	0.328
TLR6	zA489T	T/A	3.59E-11	0.20	0.18	0.18	0.18	0.88 (0.77, 1.16)	0.364	0.85 (0.64, 1.13)	0.260	0.86 (0.68, 1.10)	0.238
	T745C (rs5743810)	C/T	0.166	0.39	0.38	0.40	0.39	0.95 (0.75, 1.19)	0.641	1.05 (0.84, 1.33)	0.655	1.00 (0.82, 1.22)	0.989
	G1083C (rs3821985)	C/G	0.127	0.36	0.38	0.35	0.36	1.09 (0.87, 1.38)	0.464	0.98 (0.78, 1.24)	0.878	1.04 (0.85, 1.27)	0.731
	T1280C (rs5743815)	T/C	0.800	0.01	0.02	0.02	0.02	1.36 (0.58, 3.21)	0.480	1.10 (0.45, 2.73)	0.834	1.24 (0.67, 2.69)	0.594
	zA6852T (rs2381290)	A/T	0.775	0.49	0.47	0.46	0.46	0.95 (0.77, 1.18)	0.656	0.89 (0.71, 1.11)	0.289	0.92 (0.76, 1.11)	0.391
	T2188G	T/G	0.759	0.28	0.27	0.27	0.27	0.99 (0.78, 1.27)	0.946	1.00 (0.78, 1.27)	0.967	0.99 (0.80, 1.23)	0.950
TIRAP	zG3846A (rs125658552)	G/A	0.454	0.02	0.02	0.02	0.02	0.86 (0.39, 1.91)	0.715	1.07 (0.50, 2.29)	0.865	0.96 (0.49, 1.89)	0.911
	zA10509C (rs125665215)	A/C	0.291	0.10	0.09	0.11	0.10	0.90 (0.62, 1.30)	0.578	1.09 (0.76, 1.56)	0.633	0.99 (0.72, 1.36)	0.964
	C37T (rs8177399)	C/T	0.071	0.02	0.02	0.02	0.02	0.78 (0.33, 1.81)	0.560	1.25 (0.57, 2.66)	0.590	1.00 (0.50, 2.00)	1.000
	G164A (rs3802813)	G/A	1.000	0.05	0.05	0.05	0.05	1.07 (0.64, 1.79)	0.785	1.10 (0.66, 1.84)	0.722	1.09 (0.60, 1.70)	0.720
	G303A (rs3802814)	G/A	0.154	0.16	0.14	0.13	0.13	0.89 (0.65, 1.21)	0.446	0.80 (0.58, 1.09)	0.157	0.84 (0.64, 1.10)	0.209
	C539T (rs8177374)	C/T	1.000	0.16	0.14	0.14	0.14	0.90 (0.66, 1.21)	0.475	0.85 (0.62, 1.16)	0.296	0.87 (0.67, 1.14)	0.310
	C558T (rs7932766)	C/T	0.045	0.22	0.25	0.23	0.24	1.22 (0.94, 1.58)	0.138	1.07 (0.82, 1.40)	0.624	1.14 (0.91, 1.44)	0.253
	zT16260C (rs125670966)	T/C	1.000	0.22	0.24	0.23	0.23	1.12 (0.85, 1.45)	0.423	1.06 (0.80, 1.38)	0.701	1.09 (0.86, 1.37)	0.492
	z19085T (rs125673791)	C/T	1.000	0.11	0.11	0.12	0.12	0.96 (0.68, 1.36)	0.803	1.13 (0.80, 1.59)	0.496	1.04 (0.77, 1.40)	0.806
TRIF	zC5497T	C/T	0.101	0.07	0.08	0.08	0.08	1.11 (0.73, 1.68)	0.636	1.15 (0.76, 1.75)	0.516	1.13 (0.78, 1.63)	0.523
	zA5504G	A/G	0.240	0.15	0.18	0.18	0.18	1.19 (0.89, 1.59)	0.250	1.23 (0.91, 1.65)	0.175	1.23 (0.91, 1.57)	0.154
	zG18230T	G/T	0.007	0.16	0.16	0.15	0.15	0.99 (0.73, 1.33)	0.942	0.92 (0.68, 1.25)	0.584	0.95 (0.73, 1.24)	0.724
	C12T (rs7255265)	G/A	2.84E-14	0.39	0.36	0.38	0.37	0.89 (0.71, 1.12)	0.328	0.95 (0.76, 1.20)	0.682	0.92 (0.76, 1.12)	0.420
	C1671T (rs2292151)	C/T	0.018	0.23	0.26	0.25	0.26	1.22 (0.94, 1.57)	0.131	1.16 (0.89, 1.50)	0.272	1.19 (0.95, 1.49)	0.135

a For coding region SNPs, the name includes nucleotide numbering based on mRNA with start codon at 1. For non-coding region SNPs, the name is from the IIPGA database (http://innateimmunity.net/IIPGA2/index_html) and designated with a “z” prefix. rs numbers from the dbSNP database are included when available. A log-additive model was used for analysis. P values≤0.05 in bold.

b HWE = Hardy Weinberg Equilibrium P value, a value>0.001 indicates that polymorphism is in Hardy-Weinberg Equilibrium.

### TLR1_G1805T is associated with protection from pyelonephritis

We and others previously demonstrated that allele TLR1_1805G is associated with deficient TLR1 signaling in comparison to1805T[37], [38], [43]. We compared genotype frequencies in case and control groups and found that TLR1_1805T was associated with protection from UTIs in the combined rUTI and pyelonephritis case group in Caucasians (Table 2 log-additive model, OR(95%CI): 0.72 (0.53–0.97)). Functionally, we previously found that alleles G and T are co-dominant and that genotype 1805TT mediates higher levels of signaling than 1805GG with a mid-range level in 1805GT genotypes. In this study, we found that genotype 1805TT was associated with protection from pyelonephritis in comparison to the 1805GT and GG genotypes (Table 2, recessive model, OR(95%CI): 0.53 (0.29–0.96)). We next examined whether our results were influenced by effects of population heterogeneity. We found similar associations in the entire cohort with a protective effect of the 1805TT genotype seen when comparing controls to pyelonephritis cases (Table S1, OR(95%CI): 0.66 (0.47–0.93)).

### TLR4_A896G is associated with protection from rUTI

Allele TLR4_896G has been associated with altered signaling in response to LPS in some studies. In our study, allele 896G was associated with protection from rUTI (Table 2, log-additive model, OR(95%CI) 0.54 (0.31–0.96)), but not pyelonephritis in the Caucasian subgroup. We also examined the genotype frequencies using a dominant effect model and found that genotypes 896AG/GG were associated with protection from rUTI when compared to the 896GG genotype (Table 2, OR(95%CI): 0.54 (0.32–0.96)). A similar association was seen in the entire cohort (Table S1, log-additive model OR(95%CI) 0.60 (0.38–0.96)), These results suggest that TLR4_A896G is associated with protection from rUTI, but not pyelonephritis.

### TLR5_C1174T is associated with increased susceptibility to rUTI

We next compared genotype frequencies in case and control groups of *p*olymorphism TLR5_C1174T, which encodes a stop codon polymorphism that abrogates flagellin signaling. We previously demonstrated that allele T acts in a dominant fashion with respect to allele C [35]. The CT and TT gentoype frequencies of control women were 0.068 and 0.008, respectively, in comparison to women with rUTI who had frequencies of 0.122 and 0.003, respectively. These differences were statistically significant when comparing genotype frequencies with a dominant model (Table 2 dominant model, OR(95%CI): 1.81 (1.00–3.08)). A similar trend was observed in the log-additive model that did not reach statistical significance (OR(95%CI): 1.65 (0.89–3.05)). In contrast, no differences were seen when comparing 1174T frequencies in women with pyelonephritis versus controls. The entire cohort showed a similar association in the genotype analysis (Table S1 dominant model, OR(95%CI): 1.69 (1.06–2.70)). These results indicated that TLR5-deficiency is associated with increased susceptibility to rUTI, but not pyelonephritis. Together, these data provide evidence that SNPs TLR1_G1805T, TLR4_A896G, and TLR5_C1174T are associated with an altered risk of UTIs. However, the magnitude of the effect and the statistical significance were modest.

None of the other functional TLR SNPs were associated with UTI susceptibility in allelic or genotypic analyses (Table 2). We also generated haplotype tagging SNPs for TLRs 2, 4, 5, TIRAP, and TRIF from our sequencing data as well as TLR1 and 6 from public databases (Innate Immunity Programs for Genomic Applications database (http://innateimmunity.net/)). We derived 46 haplotype tagging SNPs, including the 7 functional SNPs that had been initially analyzed. We examined whether the genotype and haplotype frequencies of these polymorphisms differed in the pyelonephritis or rUTI case groups in comparison to the control group with no history of UTIs (Table 3 and Table S2). In this group of TLR pathway polymorphisms, there were occasional SNPs with associations with UTI risk, but they were no polymorphisms with known function, of a large magnitude, of high statistical significance, or clustered within single genes (Table 3 and Table S2, see SNPs TLR1_T130C, TLR4_zA11547G, and TRIF_C1671T). We also examined TLR1, TLR4, and TLR5 haplotypes to determine whether other SNPs in these genes modified the association with UTI susceptibility. We did not find any stronger associations within haplotypes to suggest additive or synergistic associations when the alleles were examined together (data not shown).

### TLR Polymorphisms & UTI Disease Intensity

We next examined whether SNPs TLR1_G1805T, TLR4_A896G, and TLR5_C1174T are associated with UTI disease intensity (expressed as the number of lifetime UTIs divided by age). Allele TLR5_1174T was associated with an increased number of rUTI episodes in the allelic (coefficient 0.29, P = 0.018) and genotypic analysis (dominant comparison, coefficient 0.31, P = 0.018) (Table 4). Allele 896G was associated with a decreased number of UTI episodes in the combined case group (coefficient -0.34, P = 0.011) and pyelonephritis group (coefficient -0.47, P = 0.033) (Table 4). Similar associations were found with the genotypic analysis (dominant comparison, combined case group coefficient -0.33 (P = 0.020) and pyelonephritis coefficient -0.50 (P = 0.027)). Together, these results suggest that SNPs TLR5_C1174T and TLR4_A896G are associated with altered UTI disease intensity as well as disease susceptibility.

**Table 4:** Analysis of 3 Functional TLR SNPs with UTI Disease Intensity.

SNP	group	Log-additive Model		Recessive Model^c^		Dominant Model	
		Coefficient (stdev)^a^	P^d^	coefficient (stdev)^b^	P	coefficient (stdev)^b^	P
TLR1	RUTI	0.10 (0.06)	0.102	0.09 (0.10)	0.398	0.14 (0.09)	0.113
G1805T	Pyelo	0.23 (0.11)	**0.034**	0.25 (0.19)	0.185	0.30 (0.16)	**0.053**
	Combined	0.16 (0.06)	**0.009**	0.15 (0.10)	0.160	0.22 (0.09)	**0.012**
TLR4	RUTI	−0.16 (0.16)	0.321	−0.54 (0.61)	0.373	−0.16 (0.16)	0.312
A896G	Pyelo	−0.47 (0.22)	**0.033**	−2.51 (1.43)	0.080	−0.50 (0.22)	**0.027**
	Combined	−0.34 (0.13)	**0.011**	−1.23 (0.69)	0.070	−0.33 (0.14)	**0.020**
TLR5	RUTI	0.29 (0.12)	**0.018**	0.39 (0.61)	0.521	0.31 (0.13)	**0.018**
C1174T	Pyelo	−0.09 (0.25)	0.715	−3.03 (1.42)	**0.034**	0.00 (0.26)	0.995
	Combined	0.12 (0.13)	0.361	−0.78 (0.68)	0.247	0.16 (0.13)	0.235

a Coefficient represents increase/decrease (positive/negative) in number of lifetime UTIs divided by age for each variant allele.

b Coefficient represents the difference in number of lifetime UTIs divided by age between the comparison groups.

c Recessive model comparison is unreliable due to small numbers of minor allele homozygotes.

d P values≤0.05 in bold.

## Discussion

In these analyses, we examined whether polymorphisms from the TLR pathway are associated with susceptibility to serious and recurrent UTI. The major findings of our study were that SNP TLR5_C1174T was associated with increased susceptibility to RUTI, SNP TLR4_A896G was associated with protection from RUTI, and SNP TLR1_G1805T was associated with protection from pyelonephritis. There are several possible mechanisms by which TLRs might affect the pathogenesis of cystitis and pyelonephritis. If bladder and kidney epithelial cells have a TLR-mediated signaling defect, then initial recognition of *E. coli* would be impaired and activation of signaling pathways might be delayed. In addition, neutrophils or monocytes that are recruited to the bladder might have altered responses and result in greater susceptibility to UTIs. Alternatively, or in addition, TLRs may regulate dendritic cell maturation and influence the activation and maintenance of T cell responses to *E. coli* antigens.

Previous studies have demonstrated that each of these 3 polymorphisms regulates signaling at the molecular and cellular level. SNP TLR4_A896G has been associated with decreased *in vitro* signaling in response to LPS in some studies as well as decreased *in vivo* bronchial airway responsiveness. (reviewed in [40]). Other studies did not find a difference in signaling and suggested that any functional alteration may be dependent on the assay conditions. We previously described that SNP TLR5_C1174T, which encodes a stop codon polymorphism, abolishes flagellin signalling, and is associated with increased susceptibility to Legionnaires' Disease [35]. More recently, we and others discovered that SNP TLR1_T1805G is associated with deficient TLR1 signaling as well as susceptibility to leprosy and leprosy reversal reaction [37], [38], [41], [43]. Cells from 1805TT individuals secrete 5–10 fold greater amounts of IL-6 than 1805GG cells when stimulated with PAM3, a TLR1 ligand. Intriguingly, Johnson et al also found that the TLR1 signaling defect is due to a complete absence of TLR1 on the surface of monocytes in GG individuals [38]. Recent studies have found associations with tuberculosis and sepsis [43], [49]. Greater than 80% of UTIs are caused by *E. coli* with other pathogens including *Staphylococcus saprophyticus*, and *Proteus*, *Enterobacter*, *Klebsiella*, *Pseudomonas*, and *Enterococcus* species. Each of these pathogens are known or predicted to contain cell wall lipopeptides, which are the classical ligands for TLR1-TLR2 activation.

Although numerous studies have consistently documented several behavioral risk factors for acquiring urinary tract infections, accumulating evidence suggests that genetic factors are also important. Aside from associations of non-secretor blood group antigens and P1 phenotype with RUTI and/or pyelonephritis, we are not aware of any other associations of polymorphisms with UTIs in adults [11], [12], [13], [14], [15], [16]. Several studies to date have reported associations of polymorphisms with UTI susceptibility in children. Two separate studies reported associations of CXCR1 variants with pyelonephritis [17], [18]. Karoly et al found an association of SNP TLR4 A896G with an increased risk of UTI in Hungarian children (103 cases and 235 controls) and Tabel et al found that TLR2 SNP G2258A (R753Q) was associated with UTI risk in Turkey (124 cases and 116 controls) [19], [20]. In addition, reduced levels of TLR4 expression on neutrophils was found in women with asymptomatic bacteriuria in comparison to controls [22]. Our TLR4 findings showed an opposite effect in comparison to the Karoly study. Possible explanations for the lack of confirmation of the TLR2 and TLR4 findings in our study include differences in age (pediatric vs adult), ethnicity, and polymorphism frequency (frequency of G2258A was 2% in our population in comparison to 5–13% in Turkey). It is intriguing that two genetic UTI studies have identified associations of TLRs involved in lipopeptide recognition (TLR2 and TLR1) and independently suggest an important role for this pathway in UTI pathogenesis.

Our study has several strengths and weaknesses. Potential weaknesses include effects of population admixture and multiple comparisons. To account for possible confounding effects from population heterogeneity, we performed our analyses in the Caucasian subgroup as well as the entire cohort. In addition, we enrolled our control participants from the same defined population as the cases, thus minimizing biases that might arise from a more opportunistic source of controls. Despite these attempts to minimize population admixture effects, we cannot exclude this possibility. For example, recent studies of the ancestry of European Americans indicates population substructures that could be a source of admixture [50]. This issue can be most convincingly addressed by validation of these findings in an independent study. A second possible limitation is the issue of multiple comparisons. SNPs with well-characterized function do not generally have the same requirement for adjustments for multiple comparisons due to a well-founded *a priori* hypothesis of their potential association with a cellular function. As a matter of hypothesis testing, we prioritized seven well-characterized functional polymorphisms in our primary analysis. From these seven tests, we identified three significant associations and arguably no adjustments are necessary. As a secondary analysis, we genotyped an additional 39 SNPs to determine whether haplotypes containing the 7 functional SNPs were associated with outcome. If a strict Bonferroni correction were taken, none of them would survive adjustment for multiple comparisons after multiplying the observed P values by seven or 46. However, a Bonferroni correction is arguably too stringent in this context. Although haplotype tagging polymorphisms are deliberately selected to have low levels of pairwise linkage disequilibrium, we also included additional SNPs identified in the sequencing of the coding region. The SNPs within each gene had low levels of linkage disequilibrium and were thus not fully independent tests. An alternative adjustment would be to use a False Discovery Rate method which estimates the likelihood that our findings are false. By this method, the chance of falsely observing three significant tests out of seven is quite low at approximately 11.6% (7×0.05/3). Regardless of which adjustments are chosen for our data, convincing evidence of a genetic effect ultimately requires multiple replication studies as well as detailed analysis of functional effects of each polymorphism.

Our study also had numerous strengths. First, to our knowledge this is the largest study to examine gene polymorphisms and UTI susceptibility and is the only study to date to examine this phenotype in adults. Second, to avoid a possible bias from age, we frequency matched controls to cases by age group to insure that there was similar exposure time for the development of UTIs. Finally, we enrolled subjects in a well-characterized population and verified diagnoses identified through automated indices with a clinic visit to collect additional clinical history.

Our findings raise several mechanistic questions about the role of different TLRs in UTI pathogenesis and how their effect differs for cystitis and pyelonephritis risk. Expression of individual TLRs varies in different genitourinary tissues. We previously demonstrated in a mouse model that TLR5 plays a crucial role in host defense to uropathogenic *E. coli* by limiting bacterial replication in both the bladder and kidney [30]. Other investigators have demonstrated that TLR4-deficient C3H/HeJ mice exhibit a reduced inflammatory response to *E. coli* and exhibit significantly higher bacterial counts in the bladder and kidneys [31], [51], [52]. Although TLR4 is expressed on urinary epithelium, there is conflicting evidence about whether the epithelial cells respond to LPS [53], [54], [55], [56], [57]. We found that the murine bladder was highly responsive to *in vivo* injection of flagellin, but relatively unresponsive to highly purified LPS [30]. These experiments suggest that TLR5 and TLR4 mediate distinct bladder innate immune responses with TLR5 regulating a relatively dominant role initially.

In summary, our results suggest that well-characterized polymorphisms in TLRs 1, 4, and 5 are associated with altered risks of UTI in adult women. Our work, along with others, contributes to an increased understanding of the importance of genetic variants that, along with behavioral risk factors, influence UTI susceptibility.

## Supporting Information

Table S1 (0.07 MB DOC)

Table S2 (0.06 MB DOC)
