# Supplementary material for: Toll-Like Receptor Polymorphisms and Susceptibility to Urinary Tract Infections in Adult Women
Source: PLoS One. 2009 Jun 22;4(6):e5990. doi: 10.1371/journal.pone.0005990 (PMC2696082; doi:10.1371/journal.pone.0005990)
Supplement: Table S1 — (0.07 MB DOC) [file pone.0005990.s001.doc]

**Table S1: Genotypic Analysis of Functional TLR SNPs in Whole Cohort**

|  |  | genotype | | | Log-additive Model | | Recessive Model  11 vs 00, 01 | | Dominant Model  01, 11 vs 00 | |
| --- | --- | --- | --- | --- | --- | --- | --- | --- | --- | --- |
| SNP | group | 00 | 01 | 11 | OR (95% CI) | *P^a^* | OR (95%CI) | *P* | OR (95%CI) | *P* |
| TLR1 | Control | 144 (37.0) | 136 (35.0) | 109 (28.0) |  |  |  |  |  |  |
| G1805T | rUTI | 157 (40.2) | 141 (36.1) | 93 (24.0) | 0.86 (0.71, 1.05) | 0.143 | 0.80 (0.58, 1.11) | 0.177 | 0.88 (0.66, 1.17) | 0.369 |
|  | Pyelo | 138 (39.2) | 142 (40.3) | 72 (20. 5) | 0.82 (0.67, 1.01) | 0.059 | 0.66 (0.47, 0.93) | **0.017** | 0.91 (0.68, 1.23) | 0.540 |
|  | Combined | 295 (39.7) | 283 (38.1) | 165 (22.1) | 0.84 (0.671, 1.00) | 0.052 | 0.73 (0.55, 0.97) | **0.030** | 0.89 (0.69, 1.15) | 0.379 |
| TLR2 | Control | 409 (95.3) | 20 ( 4.7) | 0 |  |  |  |  |  |  |
| G2258A | rUTI | 412 (96.0) | 17 ( 4.0) | 0 | 0.85 (0.44, 1.63) | 0.619 |  |  | 0.84 (0.44, 1.65) | 0.615 |
|  | Pyelo | 380 (95.2) | 19 ( 4.8) | 0 | 1.02 (0.54, 1.93) | 0.947 |  |  | 1.02 (0.54, 1.95) | 0.946 |
|  | Combined | 792 (95.7) | 36 ( 4.4) | 0 | 0.93 (0.54, 1.62) | 0.798 |  |  | 0.93 (0.53, 1.63) | 0.796 |
| TLR4 | Control | 379 (89.2) | 40 ( 9.4) | 6 ( 1.4) |  |  |  |  |  |  |
| A896G | rUTI | 395 (92.7) | 29 ( 6.8) | 2 ( 0.5) | 0.60 (0.38, 0.96) | **0.031** | 0.33 (0.07, 1.64) | 0.175 | 0.65 (0.40, 1.04) | 0.073 |
|  | Pyelo | 349 (88.1) | 46 (11.7) | 1 ( 0.3) | 1.01 (0.67, 1.53) | 0.954 | 0.18 (0.02,1.48) | 0.110 | 1.11 (0.72, 1.71) | 0.627 |
|  | Combined | 743 (90.5) | 75 ( 9.1) | 3 ( 0.4) | 0.80 (0.55, 1.15) | 0.226 | 0.26 (0.06, 1.03) | 0.055 | 0.87 (0.59, 1.27) | 0.460 |
| TLR4 | Control | 385 (89.7) | 40 ( 9.3) | 4 ( 0.9) |  |  |  |  |  |  |
| C1196T | rUTI | 399 (92.8) | 31 ( 7.2) | 0 | 0.63 (0.40, 1.00) | 0.051 |  |  | 0.68 (0.42, 1.10) | 0.115 |
|  | Pyelo | 354 (88.7) | 45 (11.2) | 0 | 1.01 (0.66, 1.53) | 0.969 |  |  | 1.11 (0.72, 1.73) | 0.635 |
|  | Combined | 753 (90.8) | 76 ( 9.2) | 0 | 0.81 (0.56, 1.18) | 0.268 |  |  | 0.88 (0.60, 1.04) | 0.533 |
| TLR5 | Control | 397 (92.7) | 28 ( 6.5) | 3 ( 0.7) |  |  |  |  |  |  |
| C1174T | rUTI | 379 (88.3) | 48 (11.2) | 2 ( 0.5) | 1.56 (1.00, 2.43) | **0.049** | 0.66 (0.11, 3.99) | 0.654 | 1.69 (1.06, 2.70) | **0.029** |
|  | Pyelo | 366 (91.7) | 32 ( 8.0) | 1 ( 0.3) | 1.08 (0.66, 1.75) | 0.768 | 0.36 (0.04, 3.44) | 0.373 | 1.16 (0.69, 1.92) | 0.580 |
|  | Combined | 745 (90.0) | 80 ( 9.7) | 3 ( 0.4) | 1.32 (0.88, 1.99 | 0.175 | 0.52 (0.10, 2.56) | 0.418 | 1.43 (0.93, 2.19) | 0.105 |
| TIRAP | Control | 330 (77.3) | 87 (20.4) | 10 ( 2.3) |  |  |  |  |  |  |
| C539T | rUTI | 337 (78.6) | 82 (19.1) | 10 ( 2.3) | 0.94 (0.71, 1.26) | 0.685 | 1.00 (0.41, 2.42) | 0.992 | 0.93 (0.67, 1.28) | 0.654 |
|  | Pyelo | 309 (77.8) | 82 (20.7) | 6 ( 1.5) | 0.94 (0.70, 1.26) | 0.669 | 0.64 (0.23, 1.78) | 0.392 | 0.97 (0.70, 1.34) | 0.850 |
|  | Combined | 646 (78.2) | 164 (19.9) | 16 ( 1.9) | 0.94 (0.73, 1.21) | 0.626 | 0.82 (0.37, 1.83) | 0.634 | 0.95 (0.72, 1.25) | 0.707 |
| TIRAP | Control | 277 (65.3) | 131 (30.9) | 16 ( 3.8) |  |  |  |  |  |  |
| C558T | rUTI | 258 (61.6) | 143 (34.1) | 18 ( 4.3) | 1.14 (0.90, 1.45) | 0.275 | 1.14 (0.58, 2.28) | 0.700 | 1.18 (0.89, 1.56) | 0.258 |
|  | Pyelo | 254 (64.1) | 124 (31.3) | 18 ( 4.6) | 1.06 (0.83, 1.36) | 0.618 | 1.21 (0.61, 2.42) | 0.580 | 1.05 (0.70, 1.40) | 0.722 |
|  | Combined | 512 (62.8) | 267 (32.8) | 36 ( 4.4) | 1.10 (0.90, 1.36) | 0.355 | 1.18 (0.65, 2.15) | 0.592 | 1.11 (0.87, 1.43) | 0.384 |

^a^ P-value<0.05 is in bold.
