# Supplementary material for: Toll-Like Receptor Polymorphisms and Susceptibility to Urinary Tract Infections in Adult Women
Source: PLoS One. 2009 Jun 22;4(6):e5990. doi: 10.1371/journal.pone.0005990 (PMC2696082; doi:10.1371/journal.pone.0005990)
Supplement: Table S2 — (0.06 MB DOC) [file pone.0005990.s002.doc]

**Table S2: TLR Polymorphisms & Association with UTI Disease Status in Whole Cohort**

| **Gene** | **SNP** ^a^ | **Allele** | **Minor Allele Frequency** | | | | **rUTI vs Control** | | **Pyelo vs Control** | | **Combined vs Control** | |
| --- | --- | --- | --- | --- | --- | --- | --- | --- | --- | --- | --- | --- |
|  |  |  | **Control**  **(430)** | **RUTI**  **(431)** | **Pyelo**  **(400)** | **Combined (831)** | **OR, 95% CI** | **P** | **OR, 95% CI** | **P** | **OR, 95% CI** | **P** |
| TLR1 | zC6003T  zC6165T(rs5743594)  T130C  G239C(rs5743611)  A743G (rs4833095)  G1805T (rs5743618) | T/C  C/T  T/C  G/C  A/G  G/T | 0.19 0.16 0.01 0.07 0.35 0.46 | 0.19 0.17 0.01 0.07 0.321  0.42 | 0.17 0.16 0.00 0.08 0.31 0.41 | 0.18  0.17  0.00  0.07  0.32  0.41 | 1.01 (0.79, 1.29) 1.15 (0.89, 1.49) 0.90 (0.36, 2.22) 0.91 (0.62, 1.33) 0.88 (0.72, 1.08) 0.86 (0.71, 1.05) | 0.926 0.294 0.814 0.629 0.207  0.143 | 0.91 (0.71, 1.17) 1.10 (0.84, 1.43)  0.11 (0.01, 0.84)  1.07 (0.73, 1.55) 0.85 (0.69, 1.05)  0.82 (0.67, 1.01) | 0.467 0.496  **0.033**  0.740 0.124  0.059 | 0.96 (0.78, 1.19)  1.12 (0.90, 1.41)  0.52 (0.21, 1.24) 0.99 (0.71, 1.36)  0.86 (0.72, 1.03) 0.84 (0.671, 1.00) | 0.723  0.316 0.140 0.926 0.105 **0.052** |
| TLR2 | zT540A (rs4696480)  C597T (rs3804099)  T1350C (rs3804100)  C1892A (rs5743704)  G2258A (rs5743708)  zG1868A (rs1898830) | T/A  T/C  T/C  C/A  G/A  A/G | 0.47  0.42  0.09  0.03  0.02 0.36 | 0.51  0.45  0.12  0.03  0.02 0.35 | 0.47 0.43  0.09 0.03 0.02 0.36 | 0.49  0.44  0.10  0.03  0.02  0.36 | 1.17 (0.97, 1.41)  1.13 (0.94, 1.37)  1.34 (0.98, 1.83)  1.18 (0.67, 2.07)  0.85 (0.44, 1.63) 0.95 (0.64, 1.41) | 0.109  0.202  0.069  0.567  0.619  0.803 | 1.02 (0.84, 1.23) 1.04 (0.85, 1.26) 1.01 (0.72, 1.41) 1.07 (0.60, 1.93) 1.02 (0.54, 1.93) 0.99 (0.69, 1.43) | 0.870 0.703  0.960 0.814 0.947  0.974 | 1.09 (0.93, 1.29) 1.09 (0.92, 1.28)  1.18 (0.89, 1.56) 1.13 (0.68, 1.86)  0.93 (0.54, 1.62)  0.98 (0.71, 1.34) | 0.294  0.331  0.258  0.638  0.798  0.878 |
| TLR4 | zA11547G  zG11912T (rs2149356)  zG11995A  T315C (rs5030710)  A896G (rs4986790)  C1196T (rs4986791)  GP3124C  zA17923C | A/G  G/T  G/A  T/C  A/G  C/T  G/C  C/A | 0.08  0.31  0.28  0.01  0.06  0.06  0.15  0.09 | 0.10  0.30  0.27  0.01  0.04  0.04  0.15  0.12 | 0.12  0.32  0.29  0.01  0.06  0.06 0.16  0.12 | 0.11  0.31  0.28  0.01  0.05  0.05  0.15  0.12 | 1.23 (0.88, 1.71)  0.94 (0.75, 1.18)  0.96 (0.78, 1.19)  1.00 (0.37, 2.68)  0.60 (0.38, 0.96)  0.63 (0.40, 1.00)  1.00 (0.77, 1.30)  1.28 (0.93, 1.76) | 0.227  0.619  0.719  0.996  **0.031**  0.051  0.996  0.130 | 1.58 (1.41, 2.18) 1.05 (0.83, 1.31) 1.05 (0.85, 1.30) 0.82 (0.28, 2.38) 1.01 (0.67, 1.53)  1.01 (0.66, 1.53) 0.87 (0.66, 1.14) 1.34 (0.94, 1.90) | **0.006**  0.700 0.652 0.719 0.954  0.969  0.307  0.106 | 1.39 (1.04, 1.86) 0.99 (0.82, 1.21) 1.00 (0.84, 1.21) 0.92 (0.38, 2.19)  0.80 (0.55, 1.15)  0.81 (0.56, 1.18)  0.94 (0.74, 1.18)  1.31 (0.97, 1.77) | 0.024  0.943  0.968  0.842  0.226  0.268  0.570  0.080 |
| TLR5 | C541A  C1174T (rs5744168)  A1775G (rs2072493)  T1846C (rs5744174)  A2254G  zA35403G (rs1053954) | C/A  C/T  A/G  T/C  A/G  A/G | 0.04  0.04 0.17  0.35  0.00 0.09 | 0.06 0.06 0.18 0.36 0.00 0.10 | 0.05  0.04 0.16  0.39  0.00  0.12 | 0.05  0.05  0.16  0.37  0.00  0.11 | 1.49 (0.94, 2.37)  1.56 (1.00, 2.43) 1.06 (0.83, 1.37) 1.02 (0.84, 1.25)  1.00 (0.06, 16.0)  1.14 (0.82, 1.58) | 0.092  **0.049**  0.638  0.845  1.000  0.452 | 1.14 (0.70, 1.87) 1.08 (0.66, 1.75) 0.93 (0.71, 1.21)  1.19 (0.98, 1.46)  1.08 (0.07, 17.2)  1.37 (0.99, 1.90) | 0.595  0.768 0.571  0.085  0.959  0.056 | 1.32 (0.87, 2.01)  1.32 (0.88, 1.99)  1.00 (0.80, 1.24)  1.10 (0.93, 1.31)  1.04 (0.09, 11.4)  1.25 (0.94, 1.66) | 0.195  0.175  0.974  0.275  0.978  0.130 |
| TLR6 | zA489T  T745C (rs5743810)  G1083C (rs3821985)  T1280C (rs5743815)  zA6852T (rs2381290)  T2188G | T/A  C/T  C/G  T/C  A/T  T/G | 0.19 0.30 0.39 0.01 0.43 0.22 | 0.18 0.31 0.39 0.02 0.43 0.23 | 0.17  0.34  0.36  0.02 0.42  0.23 | 0.18  0.33  0.38  0.03  0.42  0.23 | 0.93 (0.73, 1.19) 1.06 (0.86, 1.31) 0.97 (0.80, 1.19) 1.92 (0.89, 4.15) 1.02 (0.84, 1.24) 1.06 (0.85, 1.33) | 0.585  0.608  0.777 0.098  0.835  0.600 | 0.86 (0.67, 1.11) 1.19 (0.96, 1.47) 0.88 (0.72, 1.08) 1.85 (0.84, 4.07) 0.96 (0.79, 1.17) 1.07 (0.85, 1.35) | 0.239  0.107 0.214 0.125  0.689 0.570 | 0.90 (0.73, 1.11) 1.12 (0.93, 1.35) 0.93 (0.78, 1.10)  1.88 (0.93, 3.81) 0.99 (0.84, 1.17)  1.07 (0.87, 1.30) | 0.321  0.222  0.385  0.078  0.921  0.529 |
| TIRAP | zG3846A (rs125658552)  zA10509C (rs125665215)  C37T (rs8177399)  G164A (rs3802813)  G303A (rs3802814)  C539T (rs8177374)  C558T (rs7932766)  zT16260C (rs125670966)  z19085T (rs125673791) | G/A  A/C  C/T  G/A  G/A  C/T  C/T  T/C  C/T | 0.02 0.10 0.02 0.06  0.13 0.13  0.19 0.28  0.10 | 0.01 0.10 0.01  0.08 0.12 0.12 0.21 0.28  0.11 | 0.02 0.11  0.02  0.07  0.11 0.12  0.20 0.26  0.12 | 0.02  0.10  0.02  0.07  0.11  0.12  0.21  0.27  0.13 | 0.86 (0.39, 1.87)  0.95 (0.69, 1.31) 0.76 (0.33, 1.75) 1.30 (0.90, 1.88) 0.93 (0.69, 1.24) 0.94 (0.71, 1.26) 1.14 (0.90, 1.45) 0.97 (0.78, 1.20) 1.01 (0.75, 1.38) | 0.698  0.756  0.523  0.160  0.602  0.685  0.275  0.783  0.928 | 1.16 (0.56, 2.42) 1.07 (0.78, 1.47) 1.32 (0.63, 2.77) 1.02 (0.69, 1.51) 0.86 (0.64, 1.16)  0.94 (0.70, 1.26) 1.06 (0.83, 1.36) 0.90 (0.72, 1.12)  1.17 (0.86, 1.59) | 0.690  0.682 0.460  0.919 0.331  0.669  0.618 0.329  0.307 | 1.00 (0.52, 1.92) 1.01 (0.77, 1.33)  1.03 (0.53, 2.02) 1.16 (0.84, 1.62) 0.89 (0.69, 1.15) 0.94 (0.73, 1.21) 1.10 (0.90, 1.36)  0.93 (0.77, 1.13) 1.09 (0.83, 1.42) | 0.993  0.959  0.929  0.366  0.388  0.626  0.355  0.475  0.527 |
| TRIF | zC5497T  zA5504G  zG18230T  C12T (rs7255265)  C1671T (rs2292151) | C/T  A/G  G/T  G/A  C/T | 0.09 0.17 0.13 0.36 0.24 | 0.08 0.20 0.14 0.34 0.29 | 0.09 0.19 0.12  0.36  0.27 | 0.09  0.19  0.13  0.35  0.28 | 0.90 (0.64, 1.28) 1.23 (0.96, 1.57) 1.08 (0.82, 1.43) 0.88 (0.72, 1.07) 1.24 (1.00, 1.54) | 0.567  0.103  0.593  0.197  **0.052** | 1.11 (0.79, 1.56) 1.12 (0.87, 1.44) 0.94 (0.70, 1.26) 0.94 (0.77, 1.15) 1.13 (0.91, 1.41) | 0.545 0.377 0.687 0.542 0.274 | 1.00 (0.75, 1.35) 1.18 (0.95, 1.46)  1.01 (0.79, 1.29) 0.91 (0.76, 1.08)  1.18 (0.98, 1.44) | 0.989  0.144  0.922  0.266  0.078 |

^a^ For coding region SNPs, the name includes nucleotide numbering based on mRNA with start codon at 1. For non-coding region SNPs, the name is from the IIPGA database (<http://innateimmunity.net/IIPGA2/index_html>) and designated with a ‘z” prefix. rs numbers from the dbSNP database are included when available. A log-additive model was used for analysis. P values ≤ 0.05 in bold.
